# Radio frequency spectral characterization and model parameters extraction of high Q optical resonators

**DOI:** 10.1038/srep27208

**Published:** 2016-06-02

**Authors:** Zeina Abdallah, Yann G. Boucher, Arnaud Fernandez, Stéphane Balac, Olivier Llopis

**Affiliations:** 1 LAAS-CNRS, Université de Toulouse, CNRS, 7 avenue de Colonel Roche, 31031 Toulouse, France; 2 CNES, 18 avenue Edouard Belin, 31400 Toulouse, France; 3 FOTON CNRS, UMR 6082, ENSSAT, CS 80518, 22305 Lannion, France; 4 ENIB, CS 73862, F-29238 BREST cedex 3, France; 5 Univ de Toulouse, UPS, LAAS, F-31400 Toulouse, France; 6 IRMAR, Université de Rennes 1, CNRS, Campus de Beaulieu, 35042 Rennes, France

## Abstract

A microwave domain characterization approach is proposed to determine the properties of high quality factor optical resonators. This approach features a very high precision in frequency and aims to acquire a full knowledge of the complex transfer function (amplitude and phase) characterizing an optical resonator using a microwave vector network analyzer. It is able to discriminate between the different coupling regimes, from the under-coupling to the selective amplification, and it is used together with a model from which the main resonator parameters are extracted, i.e. coupling factor, intrinsic losses, phase slope, intrinsic and external quality factor.

High quality (*Q*) factor optical resonators^1^,^2^ (also known as resonant optical cavities) with different configurations, have been extensively investigated in the last decades, because of their importance in different applications such as narrow linewidth laser stabilization^3^, microwave photonics^4^, optical frequency combs generation^5^, optical filters^6^,^7^ and sensors^8^. Many different resonator technologies can be involved in these applications, with some of which featuring a ultra-high Q factors, such as: Ultra Low Expansion (ULE) Fabry-Perot resonators^9^ (*Q* ~ 10^10^), Whispering Gallery Modes (WGM) resonators, e.g. silica spheres^10^ (*Q* ~ 10^9^) or polished monocrystalline disks^11^ (*Q* ~ 10^8^ to 10^10^), active or passive fiber ring resonator^12^ (*Q* ~ 10^8^ to 10^10^). Therefore, a detailed knowledge of their properties is essential to accurately model these devices and thus to achieve better performance.

The optical resonator should be experimentally characterized in order to determine its real parameters, especially its free spectral range (*FSR*), full-width at half-maximum (Δ*f*_1/2_), loaded optical quality factor (*Q*), intrinsic quality factor (*Q*_0_), intra-cavity losses (*α*), transmission coefficient (*ρ*) and, last but not least its intra-cavity power enhancement factor (*IPEF*).

In a high *Q* factor resonator, it is difficult to induce a sufficient amount of light from conventional lasers. For example, a *Q* factor of 10^9^ near λ = 1550 nm results in a 3 dB bandwidth of 200 kHz, which is lower than the spectral width of a semiconductor laser. Using a high spectral purity laser (such as a fiber laser) can be part of the solution. However, when the laser light is injected in the resonator, it heats the resonator (even at low power, because of the photons lifetime), the resonator frequency is thus shifted and, consequently, the signal is lost.

To address these problems, several techniques have been investigated to accurately measure the resonator properties. These techniques are either based on optical domain approaches or on radio frequency domain approaches.

The first one is the optical scan method, which uses the slow tuning of a high spectral purity laser to scan the resonant modes. A good estimate of the resonator optical quality factor is provided by a slow scan of one resonant mode. However, because of the resonator’s self-heating and the resulting resonance frequency shift, the resonance plot is often different when the scan is performed with an increasing or a decreasing frequency, even if the laser frequency tuning speed is carefully adjusted^13^. This technique can thus induce errors in the measured spectral shape of ultra-high *Q* resonators, and it contains no information regarding the phase of the transfer function.

The second technique is a time domain approach based on the frequency-swept cavity ring down spectroscopy^14^,^15^,^16^, based on the measurement of the photon lifetime inside the resonant cavity by studying its relaxation regime. This technique is mostly applied to measure resonators with very high intrinsic finesse without the use of a stabilized laser. It provides not only the state of the resonator coupling regime but also the different contribution of the resonator’s parameters to its optical quality factor as well. Nevertheless, this method can only be accurate with resonator’s quality factor higher than 10^7^ and must include a least square fitting method to recover amplitude and phase transition.

The third one is a spectral domain measurement bench^17^ based on a microwave vector network analyzer (VNA) featuring a very high frequency precision as good as 1 Hz. This technique has been used to characterize several types of resonators, featuring *Q* factors ranging from 10^6^ to 10^10^. This paper demonstrates that, in addition to the amplitude response of the resonator, which gives access to the measure of several parameters like the *FSR*, the Δ*f*_1/2_ and thus the quality factor, this technique is also able to simultaneously characterize the resonator phase response thanks to a VNA. This is essential because the slope of the phase transition at the resonance is helpful to the identification of the resonator’s coupling regime, and the model extraction based on both the amplitude and the phase plot is also very accurate compared to an extraction based only on the amplitude data. In this paper, the characterization technique is thoroughly described and analyzed, and then the resonator model and the parameter extraction procedures from VNA measured data are detailed and discussed.

## Results

### Analytical study

**Figure 1:**
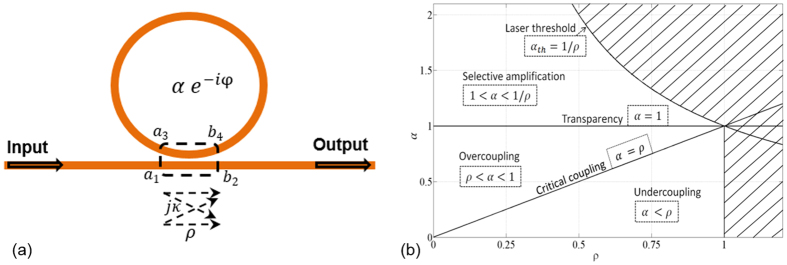
(**a**) Optical ring resonator. (**b**) Representation of the different coupling regimes as a function of *α* and *ρ*.

Temporal dependence is given as 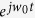. As first established by A. Yariv^18^,^19^, a simple and ideal description of an optical resonator in linear regime is depicted as monomode straight and ringing waveguides connected together through an ideal coupler (Fig. 1a). This resonator can be characterized by three essential parameters^18^,^19^: the coupler direct transmission coefficient (*ρ ∈* [0, 1]), the roundtrip attenuation or amplitude amplification coefficient (*α*, real positive number) and ring roundtrip phase (*φ*).

By varying *α* and/or *ρ* coefficient, the coupling regime is changed from the under-coupling to the selective amplification regime (Fig. 1b). If *α* < 1, the ring resonator has internal losses, this is likely to occur in the under- over- and critical regimes; if *α* > 1, the ring behaves as an amplifier and the regime is known as selective amplification. However, *α* can’t be greater than a threshold value corresponding to the system’s oscillation *α*_*th*_ = 1/*ρ*.

Using the same formalism as Yariv, which is a frequency approach based on transfer matrix, the resonator’s transfer function can be expressed as:


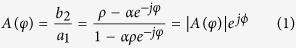


and the intensity transmission of the resonator is readily expressed from equation (1) as,


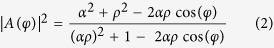


**Figure 2:**
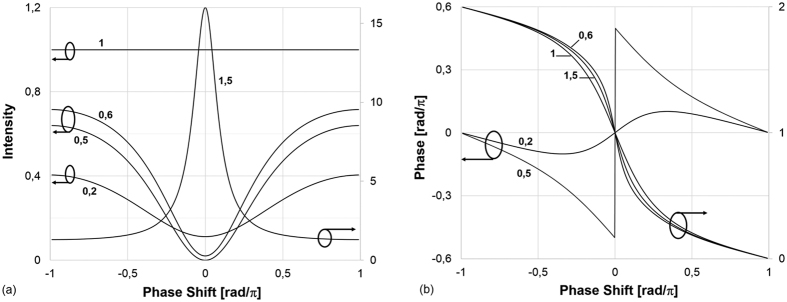
(**a**) Intensity |*A*(*φ*)|^2^ and (**b**) phase *ϕ*(*φ*) transition of the transfer function for *ρ* = 0.5 depending on the different possible coupling regime of the resonator: *α* = 0.1 (under coupling); *α* = 0.5 (critical coupling); *α* = 0.6 (over coupling); *α* = 1 (transparency); *α* = 1.5 (selective-amplification).

Previous studies^18^ have shown the intensity profile behavior as a function of the amplitude amplification parameter *α*, keeping the transmission coefficient *ρ* constant (Fig. 2a). Moreover the resonator’s *Q* factor can be deduced from the intensity profile half width (Δ*f*_1/2_) measurement thanks to formula *Q* = *ν*_0_/Δ*f*_1*/*2_, where *ν*_0_ = *w*_0_/2*π* is the optical frequency.

**Figure 3:**
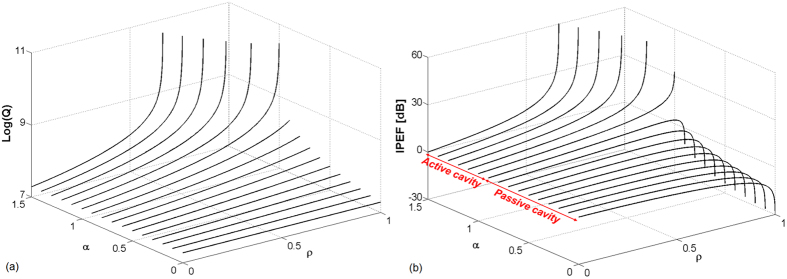
(**a**) Optical quality factor (Q) and (**b**) intra-cavity enhancement factor (*IPEF*) in function of *α* and *ρ* parameters.

Using the fact that 0 < *aρ* < 1, the exact expression of Δ*f*_1/2_ can be computed from equation (1). This yields to the following expression for the resonator’s *Q* factor:


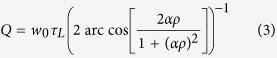


where the constant *τ*_*L*_ = *n*_*g*_*L*/*c* stands for the ring cavity roundtrip time, *n*_*g*_ is the refractive index, *L* the resonator length and *c* the speed of the light in free space. Excepted for transparency where Δ*f*_1/2_ is not defined (*A*(*φ*) = 1) because the resonator behaves as a purely dephasing component, equation (3) shows that the *Q* factor is getting higher as *aρ* increases (Fig. 3a).

In the specific case of high finesse resonators, it is usual to express the resonator’s *Q* factor in terms of the intrinsic quality factor *Q*_0_, due to the inside ring losses and corresponding to *ρ* = 1 in equation (3), and the external quality factor *Q*_*e*_ obtained from the assumption of zero losses inside the ring, i.e. *α* = 1 in equation (3), as


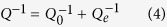


For high finesse resonators, Δ*f*_1/2_ is small and approximating cos(*φ*) by its second order Taylor expansion 1 − *φ*^2^/2 leads to the following expression for the resonator’s *Q* factor deduced from equation (3)


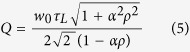


Now, to express *Q*^−1^ as a sum in which the dependence on *α* and *ρ* is separated, the second order mixed derivative of *Q*^−1^ with respect to *α* and *ρ* must be zero. This rigorously happens if and only if *αρ* = 1 but the decomposition into two separated functions can be assumed for *αρ* ≅ 1, which agrees with the high finesse assumption. Elementary differential calculus shows that the expressions of *Q*_0_ and *Q*_*e*_ are then


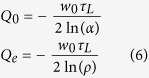


This *Q* factor expression, using *Q*_0_ and *Q*_*e*_ with separated variables, makes the link between Yariv approach and H. Haus^20^ phenomenological model.

Using Yariv formalism, the resonator’s phase *ϕ* can be deduced from equation (1) and it is expressed as:


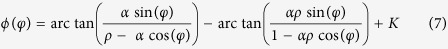


where *K* denotes a real positive constant. In under-coupling regime and at critical coupling, *K* = 0 and therefore *ϕ* ∈ [−*π*/2, *π*/2]. In selective amplification, transparency or over-coupling regime, *K* = 0 when *φ* 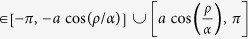, *K* = −*π* when 
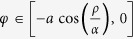
 and *K* = *π* when 
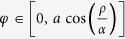
 and therefore *ϕ* ∈ [0, 2*π*] (see Fig. 2b). A particular attention has to be paid to the phase slope *F*_*D*_ given by


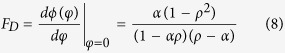


and to its sign at resonance *ϕ*(*φ* = *m*2*π*), where *m* is some integer.

The sign of *F*_*D*_, positive or negative, refers to “fast light” and “slow light” regime^21^,^22^ respectively. Note that since one can get the same intensity profile at under- and over- coupling regimes, see equation (2), the phase profile will help to discriminate each regime. It is noteworthy that *F*_*D*_ is not defined for the critical coupling since *ϕ*(*φ*) is discontinuous for *φ* = *m*2*π*.

This analytical study shows that a qualitative observation of both intensity and phase profile allows the determination of the coupling regime of the resonator and thus on the relationship between the two parameters *α* and *ρ* describing the linear resonator. Last but not least, A. Yariv’s formalism makes it possible to obtain the exact solution for the phase and intensity profiles without doing any assumption.

Additionally, it is desirable to control the intra-cavity power inside the resonator by quantifying its intra-cavity power enhancement factor (*IPEF*) as a function of *α* and *ρ* (equation 9), since due to the intra-cavity power build up at resonance, many nonlinear optical effects are generated inside the resonator, for example the stimulated Brillouin scattering, which may be harmful^12^ or beneficial^23^ depending on the application.

In the case of a passive cavity (i.e. *α* < 1), *IPEF* reaches its maximum in the critical coupling regime (*ρ* = *α*). However, in the case of an active cavity (i.e. *α* > 1), *IPEF* increases remarkably as the resonator approaches the laser oscillation threshold (*ρ* = 1/*α*_*th*_) (Fig. 3b).


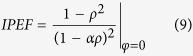


### Characterization technique description

**Figure 4:**
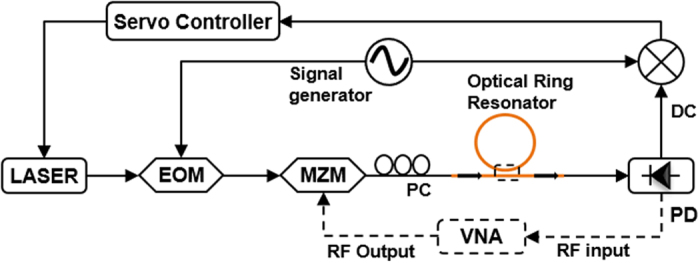
Experimental setup of the microwave characterization technique using a vector network analyzer (VNA). EOM: electro-optic modulator; MZM: Mach-Zehnder modulator; PC: polarization controller; PD: Photodiode.

The microwave characterization bench is depicted in Fig. 4. This characterization starts once the laser is stabilized onto one of the resonator’s resonant frequencies by using a low-frequency Pound-Drever-Hall (PDH) feedback loop^24^,^25^,^26^,^27^ (see section Methods for details). Other techniques may be used to lock the laser and resonator frequencies, such as thermal lock^28^ or direct optical lock, or a combination of both techniques^29^. However, these techniques are not well suited to the proposed characterization approach, firstly because the thermal lock involves a small shift between the resonator and the laser frequencies (in such a case, a single sideband modulator is needed to recover the resonator transfer function) and secondly because both techniques are not as stable as the PDH loop in case of a small room temperature drift. Also, the optical locking is only possible if a non-isolated laser is closely connected to the resonator, which is a hard to control configuration.

As soon as the laser is locked, a sweeping low power RF signal (~10 GHz) coming out of VNA’s port 1 (i.e. RF output) drives the Mach-Zehnder modulator (MZM), biased at *V*_*π*/2_ in order to get a linear modulation. The resulting modulation optical sidebands travel along an all-fiber polarization controller so as to select TE (transverse electric) or TM (transverse magnetic) modes and then go through the optical resonator. The response is finally recovered on a fast photodiode (PD), with a sufficiently large bandwidth and analyzed on port 2 of the VNA (i.e. RF input port), which displays the optical resonator’s transfer function (amplitude and phase) in electrical domain. If the laser is stabilized at the resonance center, the RF response is the optical response translated to the RF domain thanks to the frequency mixing effect of the photodiode. If the VNA has been correctly calibrated (see below), the RF losses at the resonant frequency are twice the optical losses in dB at the laser frequency (resonance center) because of the power detection effect. However, the overall slope of the transfer function is unchanged while it is brought back to the RF domain.

It is noteworthy that even though the VNA is a highly linear receiver featuring sufficient spectral purity, a number of imperfections may limit the measurement accuracy. Firstly, the laser must be properly locked to one resonance of the resonator. In case of a slightly laser detuning with resonance, the beating response of the two lateral sidebands won’t exactly reproduce the complex transfer function of the resonator. This problem can be avoided thanks to the offset function of the PDH loop. Another solution would be to use a single sideband modulator in place of a conventional MZM. This could be particularly useful in case of a dispersive resonator, for which the two lateral resonances under investigation may occur at different offset frequencies from the central one. However, this configuration is scarce on a frequency bandwidth reachable with electronic devices, i.e. below 50 GHz offset from the central resonance peak. Secondly, one must calibrate the VNA in order to measure the transfer function without taking into account the length of the access RF cables and optical fibers. This is performed by proceeding to a calibration when the resonator is bypassed. Then to cancel the remaining resonator’s pigtail fiber length (*l*), a virtual time delay *τ* is added to the calibration correction such that *τ* = *n*_*g*_*l*/*c*.

**Figure 5:**
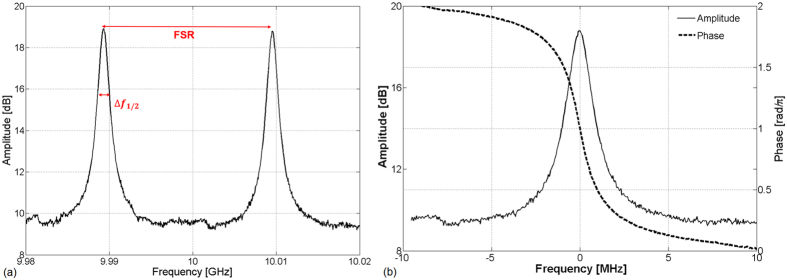
(**a**) Transfer function of an active fiber ring resonator measured using the RF-spectrum characterization bench. (**b**) A focus on one resonance in the transfer function for *P*_*Pump*_ = 21.5 *dBm*.

Figure 5a shows an example of such a measurement performed when the laser carrier is locked onto an active fiber ring resonator. This response transcribes accurately the resonator frequency comb and, focusing on the amplitude response of some resonant modes (from 9.96 to 10.02 GHz in this case), gives access to the measurement of the resonator parameters like the free spectral range (*FSR*), Δ*f*_1/2_, *Q* and the finesse *F* (defined as *FSR*/Δ*f*_1/2_).

Here, in the selective amplification regime, the measured *Q* factor is 1.66 10^8^, for *ν*_0_ and Δ*f*_1/2_ equal to 193 THz and 1.16 MHz respectively.

The profile and slope of the phase transition (Fig. 5b) at the resonance are as important as the amplitude response since they provide additional information and conditions to accurately identify the state of the resonator’s coupling regime. Along with amplitude response, they can be fitted by the analytical expressions of the transfer function (equations (2) and (7)), so as to extract the main resonator’s coefficients: *ρ* (or coupling coefficient *κ*) and *α*.

Using this technique, any problem in the characterization bench, especially in the locking process, is immediately detected via the resonance shape (amplitude of the transfer function). One of these problems could be the two states of polarization behavior of the optical carrier or a possible shift between the laser and the resonator frequencies, leading to two optical modes propagation inside the resonator. Another problem is the calibration of the VNA and particularly of the offset delay value *τ*, since an inaccurate delay could affect the measurements, precisely the slope of the phase transition of the transfer function. However, in case of high *Q* resonators characterization, the possible error on the phase slope is extremely small. As an example, a 1 cm error on a pigtail length will only lead to a relative error of 3 10^−3^ on the measured equivalent delay (and *Q* factor) of a *Q* = 10^7^ resonator. Furthermore, the bandwidth of this bench is restricted by the bandwidth of the optoelectronic components (modulator and photodiode), and in its current version, the bench is only able to characterize resonators with an *FSR* smaller than 50 GHz.

## Discussion

An experiment has been carried out to demonstrate the validity of the RF-spectrum characterization technique using an active optical fiber ring resonator^30^,^31^, which has been designed using an erbium-doped fiber amplifier and an optical bidirectional coupler (see section Methods for more details). The laser used in this experiment is a fiber laser (Koheras Adjustik) with a very narrow linewidth (1 kHz) emitting at λ = 1550 nm. The laser lock on a resonance is achieved first by varying the laser temperature and then using a piezoelectric control input. Once locked, the amplitude and the phase of the resonator’s output have been measured while varying the intra-cavity losses *α* (by changing the laser pump power, *P*_*Pump*_), in order to assess the different coupling regimes. Figure 5b depicts the transfer function (amplitude and phase) for a pump power of 21.5 dBm. From its amplitude curve and its phase slope at the resonance, it is obvious that the resonator is in selective amplification regime.

**Figure 6:**
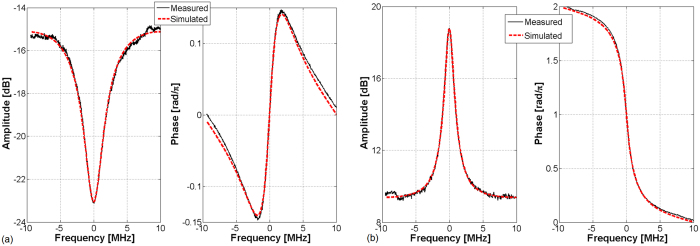
Experimental data (black solid line) and curve fitting (red dotted line) of the amplitude and phase of the output signal (**a**) *P*_*Pump*_ = 7.5 dBm, (**b**) *P*_*Pump*_ = 21.5 dBm.

The experimental data (amplitude and phase) are then fitted with equations (2) and (7) using the least mean square algorithm, which is based on only two parameters, i.e. the intra-cavity losses (or gain) *α* and the transmission coefficient *ρ*. Figure 6 shows the fitted and measured data for both the amplitude and phase of the output transfer function under two different laser pump power (7.5 and 21.5 dBm). The *α* and *ρ* parameters are thus extracted from the numerical fitting method (Table 1).

For low laser pump power (7.5 dBm, i.e. low gain amplifier, Fig. 6a), the resonator is in under-coupling regime, which means that the intra-cavity losses must be lower than the coupler transmission coefficient, i.e. *α* < *ρ*. For high *P*_*Pump*_ (21.5 dBm, Fig. 6b), precisely for 1 < *α* < 1/*ρ*, the selective amplification is obtained. Once *α* and *ρ* are obtained, the *Q*_0_ and *Q*_*e*_ factors can be calculated and *Q* values are computed from equations (3) and (4). The obtained values of the fitting parameters meet well the conditions for each coupling regime.

When the resonator is in its selective amplification regime, *α* is a gain (1 < *α* < 1/*ρ*) and the intrinsic quality factor *Q*_0_ is negative. The computed *Q* values are compared to the measured data, 3.26 10^7^ and 1.66 10^8^ for 7.5 and 21.5 dBm of laser pump current respectively, in order to obtain the error. A good agreement is found between measured and computed values of *Q* from the extracted *α* and *ρ* data.

It’s notable that even though the cavity ring down approach allows to get the same parameters, the fitting procedure in the spectrum-RF approach allows a simple and more accurate extraction of the resonators’ state and parameters. In fact, the modulus and phase transfer function are immediately observed with spectrum-RF method which is not the case with cavity ring down approach. This allows a visual and straightforward deduction of the coupling regime and of the relationship between *α* and *ρ* coefficients. This relationship is immediately fed to the least mean square algorithm as initial condition.

## Methods

### Stabilization technique description

The Pound-Drever-Hall (PDH)^24^,^25^,^26^,^27^ laser stabilization technique is based on using the phase information of a laser signal passing through the resonator. Firstly, the laser carrier is phase modulated with a low frequency (*f*_*G*_) by means of an electro-optic modulator (EOM). The signal generator frequency (*f*_*G*_) is typically a few MHz, which is around the resonator bandwidth. The transmitted signal through the resonator is then sent to a fast photodiode. The amplitude of the photodiode’s output voltage is filtered and sent to an electrical mixer to be mixed with a fraction of the low frequency signal (*f*_*G*_) used to drive the modulator. The mixer’s output represents an error signal proportional to the frequency detuning between the laser and the resonance. Finally, the error signal is sent to a servo-controller in order to adjust the laser’s wavelength.

### Active optical fiber ring resonator

**Figure 7:**
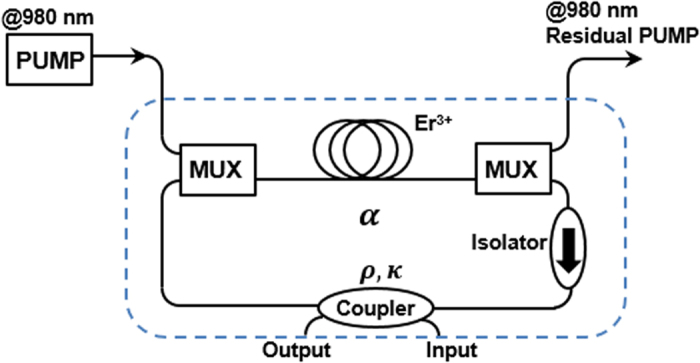
Active fiber ring resonator configuration. MUX: Multiplexing.

Figure 7 depicts the active optical resonator which was designed using a fibered 2 × 2 optical bidirectional coupler linked to a 2.1m-long erbium-doped (DrakaElite eHPW-9) fiber amplifier. The coupler’s transmission coefficient (*ρ*) is 50%. An isolator is added to prevent backward reflections and to ensure unidirectional light wave propagation. By varying the laser pump power, it is possible to change the roundtrip cavity losses (*α*) of the resonator and therefore to control the coupling regime from under-coupling to over-coupling, and up to the selective amplification.

## Additional Information

**How to cite this article**: Abdallah, Z. *et al*. Radio frequency spectral characterization and model parameters extraction of high Q optical resonators. *Sci. Rep*. **6**, 27208; doi: 10.1038/srep27208 (2016).

## Figures and Tables

**Table 1:** Fitting parameters values for *P*_*Pump*_ = 7.5 dBm and *P*_*Pump*_ = 21.5 dBm, the fitted *Q* values are computed using equations (3) and (4) and then compared with the measured value for both cases.

		***P***_***Pump***_** = 7.5 dBm**	***P***_***Pump***_** = 21.5 dBm**
*α*		0.503	1.2
*ρ*		0.72	0.69
*Q*_0_		4.69 10^7^	−1.65 10^8^
*Q*_*e*_		9.75 10^7^	8.44 10^7^
*Q*	Equation (3)	≈3.46 10^7^ (5.7% error)	≈1.66 10^8^ (3.6% error)
	Equation (4)	≈3.17 10^7^ (3% error)	≈1.72 10^8^ (3.5% error)

## References

[b1] Vahala K. J. Optical microcavities. Nature 424, 839–846 (2003). 12917698 10.1038/nature01939

[b2] Heebner J., Grover R. & Ibrahim T. Optical microresonators: theory, fabrication, and application (Springer, 2008).

[b3] Liang W. . Ultralow noise miniature external cavity semiconductor laser. Nat. Commun. 6, 1–6 (2015). 10.1038/ncomms8371 PMC4491184 26104321

[b4] Savchenkov A. A. . Whispering-gallery mode based opto-electronic oscillators. *Frequency Control Symposium (FCS), 2010 IEEE International*, Newport Beach, CA, 554–557, doi: 10.1109/FREQ.2010.5556268 (2010).

[b5] Kippenberg T. J., Holzwarth R. & Diddams S. A. Microresonator-based optical frequency combs. Science 332, 555–559 (2011). 21527707 10.1126/science.1193968

[b6] Mandal S., Dasgupta K., Basak T. K. & Ghosh S. K. A generalized approach for modeling and analysis of ring-resonator performance as optical filter. Optics Communication 264, 97–104 (2006).

[b7] Merrer P. H., Llopis O., Nicole P. & Constant S. Modeling and practical demonstration of multiple optical fiber ring resonators in the microwave domain. Microw. Opt. Technol. Lett. 54, 1552–1556 (2012).

[b8] Maxwell A. . Polymer microring resonators for high-frequency ultrasound detection and imaging. IEEE. J. Sel. Topics Quantum Electron. 14, 191–197 (2008). 10.1109/JSTQE.2007.914047 PMC2917845 20700482

[b9] Ludlow A. D. . Compact, thermal-noise-limited optical cavity for diode laser stabilization at 1 × 10^−15^. Opt. Lett. 32, 641–643 (2007). 17308587 10.1364/ol.32.000641

[b10] Righini G. C. Whispering gallery mode microresonators: Fundamentals and applications. Riv. Nuovo. Cimento 34, 437–488 (2011).

[b11] Savchenkov A., Ilchenko V., Matsko A. & Maleki L. Kilohertz optical resonances in dielectric crystal cavities. Phys. Rev. A 70, 051804 (2004).

[b12] Saleh K., Llopis O. & Cibiel G. Optical scattering induced noise in fiber ring resonators and optoelectronics oscillators. J. Lightwave Technol. 31, 1433–1446 (2013).

[b13] Merrer P. H. . Microwave filtering using high Q optical resonators. *Microwave Conference, 2008. EuMC 2008. 38*^*th*^ *European*, Amsterdam, 2008, 381–384, doi: 10.1109/EUMC.2008.4751468 (2008).

[b14] Hahn J. W., Yoo Y. S., Kim J. W. & Lee H. W. Cavity ringdown spectroscopy with a continuous-wave laser: calculation of coupling efficiency and a new spectrometer design. Appl. Opt. 38, 1859–1886 (1999). 18305817 10.1364/ao.38.001859

[b15] He Y. & Orr B. J. Ringdown and cavity-enhanced absorption spectroscopy using a continuous-wave tunable diode laser and a rapidly swept optical cavity. Chem. Phys. Lett. 319, 131–137 (2000).

[b16] Dumeige Y. . Determination of coupling regime of high-Q resonators and optical gain of highly selective amplifiers. J. Opt. Soc. Am. B 25, 2073–2080 (2008).

[b17] Merrer P. H. . Characterization technique of optical whispering gallery mode resonators in the microwave frequency domain for optoelectronic oscillators. Appl. Opt. 51, 4742–4748 (2012). 22781250 10.1364/AO.51.004742

[b18] Yariv A. Universal relations for coupling of optical power between micro-resonators and dielectric waveguides. Electronics Letters 36, 321–322 (2000).

[b19] Yariv A. Critical coupling and its control in optical waveguide-ring resonator systems. IEEE Photon. Technol. Lett. 14, 483–485 (2002).

[b20] Haus H. A. Waves and fields in optoelectronics (Prentice-hall, 1984).

[b21] Tomita M., Uesugi H., Sultana P. & Oishi T. Causal information velocity in fast and slow pulse propagation in an optical ring resonator. Phys. Rev. A 84, 043843 (2011).

[b22] Rasoloniaina A. . Controling the coupling properties of active ultrahigh-Q WGM microcavities from undercoupling to selective amplification. Sci. Rep. 4, 4023, doi: 10.1038/srep04023 (2014). 24503956 PMC3916894

[b23] Geng J. . Highly stable low-noise brillouin fiber laser with ultranarrow spectral linewidth. IEEE. Photon. Technol. Lett. 18, 1813–1815 (2006).

[b24] Black E. D. An introduction to Pound-Drever-Hall laser frequency stabilization. Am. J. Phys. 69, 79–87 (2001).

[b25] Merrer P. H., Llopis O. & Cibiel G. Laser stabilization on a fiber ring resonator and application to RF filtering. IEEE Photon. Technol. Lett. 20, 1399–1401 (2008).

[b26] Drever R. V. Electronic frequency stabilization of microwave oscillators. Rev. Sci. Instrum. 17, 490–505 (1949). 10.1063/1.1770414 20280200

[b27] Drever R. W. P. . Laser phase and frequency stabilization using an optical-resonator. Appl. Phys. B. 31, 97–105 (1983).

[b28] Carmon T., Yang L. & Vahala K. J. Dynamical thermal behavior and thermal self-stability of microcavities. Opt. Express 12, 4742–4750 (2004). 19484026 10.1364/opex.12.004742

[b29] McRae T. G., Lee K. H., McGovern M., Gwyther D. & Bowen W. P. Thermo-optic locking of a semiconductor laser to a microcavity resonance. Opt. Express 17, 21977–21985 (2009). 19997442 10.1364/OE.17.021977

[b30] Zhang F. & Lit J. W. Y. Direct-coupling single-mode fiber ring resonator. J. Opt. Soc. Am. A 5, 1347–1355 (1988).

[b31] Heebner J. E., Wong V., Schweinsberg A., Boyd R. W. & Jackson D. J. Optical transmission characteristics of fiber ring resonators. IEEE. J. Quantum Electron. 40, 726–730 (2004).

